# Impact of Sulfur Fumigation on Ginger: Chemical and Biological Evidence

**DOI:** 10.1021/acs.jafc.2c05710

**Published:** 2022-09-21

**Authors:** Wei-Hao Zhang, Han-Yan Luo, Jing Fang, Chen-Liang Zhao, Kam-Chun Chan, Yui-Man Chan, Cai-Xia Dong, Hu-Biao Chen, Zhong-Zhen Zhao, Song-Lin Li, Jun Xu

**Affiliations:** † School of Chinese Medicine, Hong Kong Baptist University, Hong Kong 999077, China; ‡ College of Pharmacy, Guizhou University of Traditional Chinese Medicine, Guiyang 550002, China; § Tianjin Key Laboratory on Technologies Enabling Development of Clinical Therapeutics and Diagnosis, School of Pharmacy, Tianjin Medical University, Tianjin 300070, China; ∥ Department of Pharmaceutical Analysis, Affiliated Hospital of Integrated Traditional Chinese and Western Medicine, Nanjing University of Chinese Medicine, Nanjing 210028, China

**Keywords:** ginger, sulfur fumigation, 6-gingesulfonic acid, 6-shogaol, systemic exposure, anticancer

## Abstract

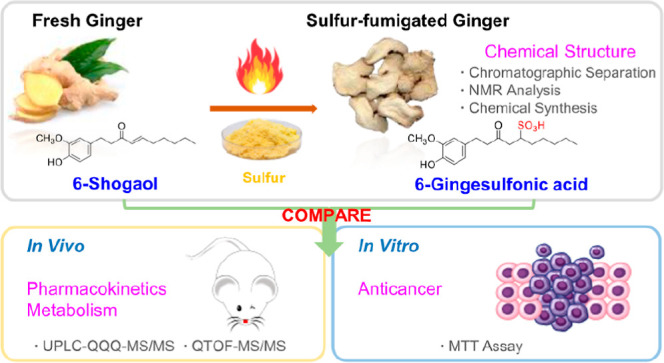

We previously found that sulfur fumigation, a commonly used controversial method for the post-harvest handling of ginger, induces the generation of a compound in ginger, which was speculated to be a sulfur-containing derivative of 6-shogaol based on its mass data. However, the chemical and biological properties of the compound remain unknown. As a follow-up study, here we report the chemical structure, systemic exposure, and anticancer activity of the compound. Chromatographic separation, nuclear magnetic resonance analysis, and chemical synthesis structurally elucidated the compound as 6-gingesulfonic acid. Pharmacokinetics in rats found that 6-gingesulfonic acid was more slowly absorbed and eliminated, with more prototypes existing in the blood than 6-shogaol. Metabolism profiling indicated that the two compounds produced qualitatively and quantitatively different metabolites. It was further found that 6-gingesulfonic acid exerted significantly weaker antiproliferative activity on tumor cells than 6-shogaol. The data provide chemical and biological evidence that sulfur fumigation may impair the healthcare functions of ginger.

## 1 Introduction

Ginger derived from the rhizome of *Zingiber officinale* Roscoe is used as flavoring all around the world.^1^ It is now associated with many health benefits, such as anticancer, antioxidant, anti-inflammatory, antimicrobial, neuroprotective, and cardiovascular protective activities.^2−4^ Shogaols are one of the major chemical types contributing to these functional activities, and 6-shogaol in particular has attracted extensive attention due to its favorable bioactivities.^2,5,6^ For example, accumulating evidence has revealed that 6-shogaol can induce cellular death and apoptosis in a variety of cancer cells including human lung cancer, colorectal carcinoma, hepatocarcinoma, ovarian cancer, and breast cancer cells.^7^

Sulfur fumigation has been used controversially in the post-harvest handling of ginger for retaining moisture, preserving color and freshness, and preventing damage from insects and moulds.^8^ However, we previously demonstrated that sulfur fumigation significantly alters the chemical profile of ginger. In particular, we found a compound with a *m*/*z* value of 357.13 (compound I) was generated in ginger by sulfur fumigation, and it was statistically screened out as the chemical marker of sulfur-fumigated ginger. According to its mass data, compound I was temporarily speculated to be a sulfur-containing derivative of 6-shogaol as the product of electrophilic addition in the presence of sulfonic acid.^9^ However, the chemical and biological properties of compound I, which is necessary to further understand the impact of sulfur fumigation on ginger, remain to be explored.

Following this research, here, we report the chemical structure, systemic exposure, and anticancer activity of compound I. First, the chemical structure of compound I was unambiguously elucidated using chromatographic separation, nuclear magnetic resonance (NMR) analysis, and chemical synthesis. Then, its pharmacokinetics and metabolism in rats were investigated and compared to 6-shogaol, and their antiproliferative effects on human cancer cell lines were also evaluated and compared.

## 2 Materials and Methods

### 2.1 Chemical Reagents, Materials, and Cell Lines

Sulfur was obtained from Sigma-Aldrich (Steinheim, Germany). 6-Shogaol (purity at 98.15%) was purchased from Nanjing Dilger Medical Technology Company (Nanjing, China). Macroporous resin D101 and Sephadex LH-20 were purchased from Macklin Biochemical Co., Ltd (Shanghai, China). Silica gel and preparative thin layer chromatography plate were provided by Qingdao Marine Chemical Factory (Qingdao, China). Methanol-d4 with 0.03% tetramethylsilane (TMS) was purchased from Shanghai Aladdin Biochemical Technology Company (Shanghai, China). Acetonitrile and methanol of HPLC grade were obtained from Fisher Co., Ltd. (Waltham, MA, USA). Formic acid of analytical grade was purchased from Beijing Chemical Reagent Company (Beijing, China). Ultrapure water was produced by a Milli-Q water purification system (Milford, MA, USA). Other solvents and chemicals were of analytical grade.

Fresh ginger was collected from a local farm (Zen Organic Farm, Ta Kwu Ling, New Territories of Hong Kong) and was authenticated as the rhizomes of *Z. officinale* Rosc. (Chinese ginger) by Dr. Jun Xu. Voucher specimens of the ginger samples were deposited at the School of Chinese Medicine, Hong Kong Baptist University.

HCT-116 colon cancer cells (Procell, Wuhan, China) were cultured in McCoy’s 5A medium (Gibco; Thermo Fisher Scientific Inc., MA, USA), Hep-G2 hepatocellular carcinoma cells (Procell, Wuhan, China) in Eagle’s minimum essential medium (EMEM, Gibco; Thermo Fisher Scientific Inc., MA, USA), and HCC-1806 breast cancer cells (Manassas, VA, USA) in RPMI-1640 medium (Gibco; Thermo Fisher Scientific Inc., MA, USA). All the mediums were supplemented with 10% fetal bovine serum and 1% penicillin–streptomycin (Thermo Fisher Scientific Inc., MA, USA) and cultured at 37 °C in a humidified incubator with 5% CO_2_.

### 2.2 Isolation and Structural Elucidation of Compound I

The sulfur-fumigated ginger sample was prepared as we previously reported.^9^ A total of 1.15 kg sulfur-fumigated ginger (dry weight) were powdered and extracted with 90% ethanol (3 × 5 L). The extracts were combined and concentrated under vacuum to give a residue, which was then suspended in H_2_O (1.5 L) and extracted with petroleum ether (3 × 2 L), ethyl acetate (3 × 2 L), and *n*-butanol (3 × 2 L), successively. Then organic solvents were evaporated under reduced pressure to provide extracts of petroleum ether (24.7 g), ethyl acetate (88.3 g), and *n*-butanol (10.8 g). Next, the ethyl acetate extracts were chromatographed on a D101 resin column eluted with EtOH–H_2_O (from 0:100 to 100:0, v/v) to furnish 18 fractions A1–A18. Then fractions A10–A15 (1.5 g) were further loaded on a silica gel column eluted with CH_2_Cl_2_–MeOH (from 100:0 to 0:100, v/v) to afford 12 fractions B1–B12. B6–B9 (497.6 mg) was subjected to a Sephadex LH-20 (100% MeOH) to yield fractions C1–C21. The preparative high-performance liquid chromatography (prep-HPLC) separation was performed on a Waters Prep 2695 LC system that included an auto-sampler (2707), a quaternary gradient module (2545), and a dual absorbance detector (2487). Data were collected using a MassLynx 2.0 workstation (Waters, USA). The separation was achieved on an Alltech Prep C18 column (19 mm × 150 mm, 5 μm) with a mobile phase consisting of (A) water and (B) acetonitrile. Compound I (8.5 mg) was finally obtained from fractions C12–C16 (159.3 mg) by prep-HPLC (15% acetonitrile). The mass spectrum information of compound I was obtained by ultra-performance liquid chromatography with a quadrupole time-of-flight mass spectrometer (UPLC–QTOF–MS/MS) (Agilent 1290 UPLC system coupled with a 6540 QTOF mass spectrometer, Agilent Technologies, Santa Clara, CA, USA). The ^1^H NMR and ^13^C NMR spectra of compound I were recorded on FT-NMR, 400 MHz (Bruker Avance-III), using methanol-d4 as the solvent and TMS as the internal standard.

### 2.3 Synthesis of Compound I

A methanol solution of 6-shogaol (20 mg/5 mL) was treated with NaHSO_3_ (74.29 mg) and *tert*-butyl peroxybenzoate (TBPB, 28.75 μL), and the mixture was stirred at 75 °C for 30 h. The solvent was removed, and the product was then purified by preparative thin-layer chromatography (prep-TLC) (CH_2_Cl_2_/MeOH = 3:1) and prep-HPLC (15% acetonitrile) to furnish compound I.

### 2.4 Systemic Exposure In Vivo

#### 2.4.1 Animals and Drug Administration

A total of 12 adult male Sprague–Dawley rats (220–240 g) were purchased from the Chinese University of Hong Kong. The animals were kept in standard living conditions (room temperature 22 ± 1 °C, 12 h light/dark normal cycle, and constant humidity) at the animal laboratory of the School of Chinese Medicine, Hong Kong Baptist University with free access to food and water. All experimental protocols were approved by the Environmental Health and Safety Committee of Hong Kong Baptist University, Hong Kong (03/2017), and the procedures were in accordance with the guidelines of the Animal Care Ethics Committee of Hong Kong Baptist University and the Department of Health, Hong Kong Special Administrative Region. Before the experiment, the rats were kept for one week to acclimate to the new environment. The rats were randomly divided into two groups (*n* = 6) and fasted with water freely available. On the day of the experiment, by intragastric administration, one group received compound I and the other group received 6-shogaol. Both compounds were dissolved in 2% Tween 80 with an administration volume of less than 0.5 mL at a dose of 10 mg/kg. Then, rats were housed singly during the blood collection period (a total of 72 h). For each sample, 300–500 μL blood was collected from the eye canthus of each rat into tubes containing 1.5 mL EDTA. Samples were drawn at 0.0, 0.08, 0.25, 0.50, 0.75, 1.0, 2.0, 4.0, 6.0, 12, 24, 36, 48, 60, and 72 h. The blood samples were centrifuged at 4000 rpm for 15 min at 4 °C to obtain plasma. Urine and feces were collected every 1–2 h throughout the experiments. The blood, urine, and feces were collected before the intragastric administration as a blank sample. The rats were sacrificed by cervical spondylolisthesis after the experiment.

#### 2.4.2 Sample Preparation

For quantitative determination of plasmatic prototype component concentrations, 50 μL plasma from each sample was added to 1.2 mL of MeOH to precipitate proteins. After centrifugation at 15,000 rpm for 15 min, the supernatants were transferred into vials for analysis. For metabolite identification and relative quantification, homogeneous biospecimen samples were combined during periods from 0 h to the time when plasmatic concentrations reached a maximum value (*T*_max_) after intragastric administration. 50 μL of each plasma or urine sample was added to 1.2 mL of MeOH to precipitate proteins. After centrifugation at 15,000 rpm for 15 min, the supernatants were mixed and transferred into vials for analysis. To prepare feces samples, 10 g fresh feces sample was put into 50 mL tubes. 10 mL of MeOH/H_2_O (50%/50%, v/v) with 0.1% acetic acid was then added to each sample. The samples were sonicated for 60 min and then centrifuged at 15,000 rpm for 15 min. The concentrated supernatant (1.5 mL) was collected and frozen out for further analysis.

#### 2.4.3 Quantitative Determination

A multiple reaction monitoring (MRM) mass acquisition method using a triple quadrupole (QQQ) mass spectrometer was used to determine compound I and 6-shogaol in rat plasma. Chromatographic separation was performed on an Agilent 1290 UPLC equipped with a binary pump with degasser (G4220A), a thermostatic column compartment (G1316C), and an autosampler (G4226A). The samples were kept at 8 °C in the autosampler. An aliquot of sample (2 μL) was injected into a Waters Acquity HSS C18 column (100 Å, 1.8 μm, 2.1 mm × 50 mm) operated at 40 °C. The mobile phase used for the UPLC consisted of 0.1% formic acid in water (v/v) (A) and 0.1% formic acid in acetonitrile (v/v) (B). The elution gradient procedure started at 15% B, then 0–5 min, 15–75% B; 5–6 min, 75–100% B; 6–9 min, 100% B; 9–12 min, 15% B.

MS data were acquired with an Agilent 6460 QQQ mass spectrometer (G6460) equipped with a JetStream electrospray ion (ESI) source. The MRM parameters were as follows: for 6-gingesulfonic acid, negative mode; fragmentor at 120 V; ion pair *m*/*z* 357.1 → 80.9 (15 V), 357.1 → 275.1 (15 V). For 6-shogaol, positive mode; fragmentor at 80 V; ion pair *m*/*z* 277.2 → 137.0 (11 V), 277.2 → 259.1 (7 V). The operating source parameters were as follows: nebulizing gas (N_2_) flow rate at 8 L/min; nebulizing gas temperature at 300 °C; JetStream gas flow at 7 L/min; sheath gas temperature at 350 °C; nebulizer pressure at 45 psi; capillary voltage at 3500 V; skimmer at 65 V; Octopole RFV at 1000 V. MS data were collected with Mass Hunter Qualitative Analysis B.06 and Quantitative Analysis B.06 software.

#### 2.4.4 Quantitative Method Validation

The established UPLC–QQQ–MS/MS methods were validated according to the guidelines of the US Food and Drug Administration.^10^ Linearity, limit of detection (LOD), lower limit of quantification (LLOQ), interday and intraday accuracy and precision, extraction recovery, and stability were measured. The calibration curve was replicated three times on the same day (intraday) and over 3 days (interday) to evaluate accuracy and precision. The correlation coefficient (*r*^2^) of all calibration curves was at least 0.995, and the bias and coefficient of variation were kept within ±15%. Matrix effect and recovery evaluation: the matrix effect was given as the ratio of mean peak area for the post-extraction spiked samples (set 2, in plasma) to that of the standard solution samples (set 1, in MeOH). Recovery was quantified as the ratio of mean peak area of the pre-extraction spiked samples (set 3, standard with homogenate plasma) to that of the post-extraction spiked samples (set 2, in plasma). Stability: (1) freeze and thaw: samples were stored at −20 °C for 24 h and thawed at room temperature and repeated three times; (2) short term: samples were maintained at room temperature for 4 h; (3) long term: samples were kept at −20 °C for 30 days; (4) postpreparative: samples were maintained in an autosampler for 8 h. The stability of all the samples was limited to within ±15%, except for the LLOQ within ±20%.

#### 2.4.5 Pharmacokinetic Examination

The pharmacokinetic parameters were calculated using the PKSolver 2.0 add-in with a non-compartment model.^11,12^ The time to peak drug concentration (*T*_max_) means the time when plasma concentrations reached a maximum value (*C*_max_). The area under the concentration versus time curve from zero to the last sampling time (AUC_0–*t*_) and to infinity (AUC_0–∞_), apparent volume of distribution (Vz/F_obs), terminal half-life (*T*_1/2_), mean residence time (MRT), and apparent total body clearance (Cl/F_obs), were calculated under the PKSolver add-in. All the results were presented as the mean ± standard deviation (SD) of six independent experiments.

#### 2.4.6 Metabolite Identification

An auto MS/MS acquisition method of UPLC–QTOF–MS/MS was used to identify the metabolites of compound I and 6-shogaol in plasma, urine, and feces. It carried on an Agilent 1290 UPLC system as mentioned. The samples were kept at 8 °C in the autosampler. An aliquot of sample (2 μL) was injected into a Waters Acquity BEH C18 column (100 Å, 1.8 μm, 2.1 mm × 100 mm) operated at 40 °C. The separation was achieved using gradient elution with 0.1% formic acid in water (A) and acetonitrile (B) at a flow rate of 0.40 mL/min. The elution program was as follows: 0–18 min, 5–75% B; 18–24 min, 75–100% B; 24–27 min, 100% B; 27–30 min, 5%.

MS data were acquired with an Agilent 6540 QTOF mass spectrometer (G6540A) equipped with a JetStream ESI source in Auto MS/MS full scan mode. The optimized operating parameters in negative ion mode were as follows: the nebulizing gas (N_2_) flow rate at 7 L/min; nebulizing gas temperature at 300 °C; JetStream gas flow at 7 L/min; sheath gas temperature at 300 °C; nebulizer pressure at 37 psi; capillary voltage at 3000 V; skimmer at 65 V; Octopole RFV at 600 V; and fragmentor voltage at 180 V. The mass spectra were recorded from 100 to 1700 *m*/*z* with accurate mass measurement of all mass peaks; the scanning range of MS/MS was from 50 to 1700 *m*/*z*. Metabolites were analysed using Agilent Mass Hunter (including qualitative analysis and quantitative analysis) and Mass Hunter Profiler B.02 software.

### 2.5 Evaluation of Antiproliferative Effects on Human Cancer Cells

Cell viability was determined by a 3-(4,5-dimethylthiazol-2-yl)-2,5-diphenyltetrazolium bromide (MTT) colorimetric assay. Each cell strain (6000 cells/well) was plated in 96-well microtiter plates and allowed to attach for 24 h at 37 °C and 5% CO_2_. Compound I or 6-shogaol (in dimethyl sulfoxide, DMSO) was added to the cell culture medium to desired final concentrations of 10, 40, and 100 μM. Final DMSO concentrations for control and treatments were 0.1%. There were six replicates of each condition. After the cells were cultured for 24 h, the medium was removed, and the cells were treated with 5 mg/mL MTT in fresh media. After incubation for 4 h at 37 °C, the medium containing MTT was removed, 100 μL of DMSO was added to the wells, and the plates were shaken gently for 30 min at room temperature. Absorbance values were derived from the plate reading at 490 nm on microplate readers (BioTek, Agilent).

### 2.6 Statistical Analysis

Experimental results were presented as means of repeated experiments ± SD. Data analyses were computed using GraphPad Prism (version 9.1.0). Differences between the groups were evaluated by the student’s *t*-test and comparisons that yielded *p* values <0.05 were considered significant.

## 3 Results and Discussion

### 3.1 Structural Elucidation of Compound I

**Figure 1:**
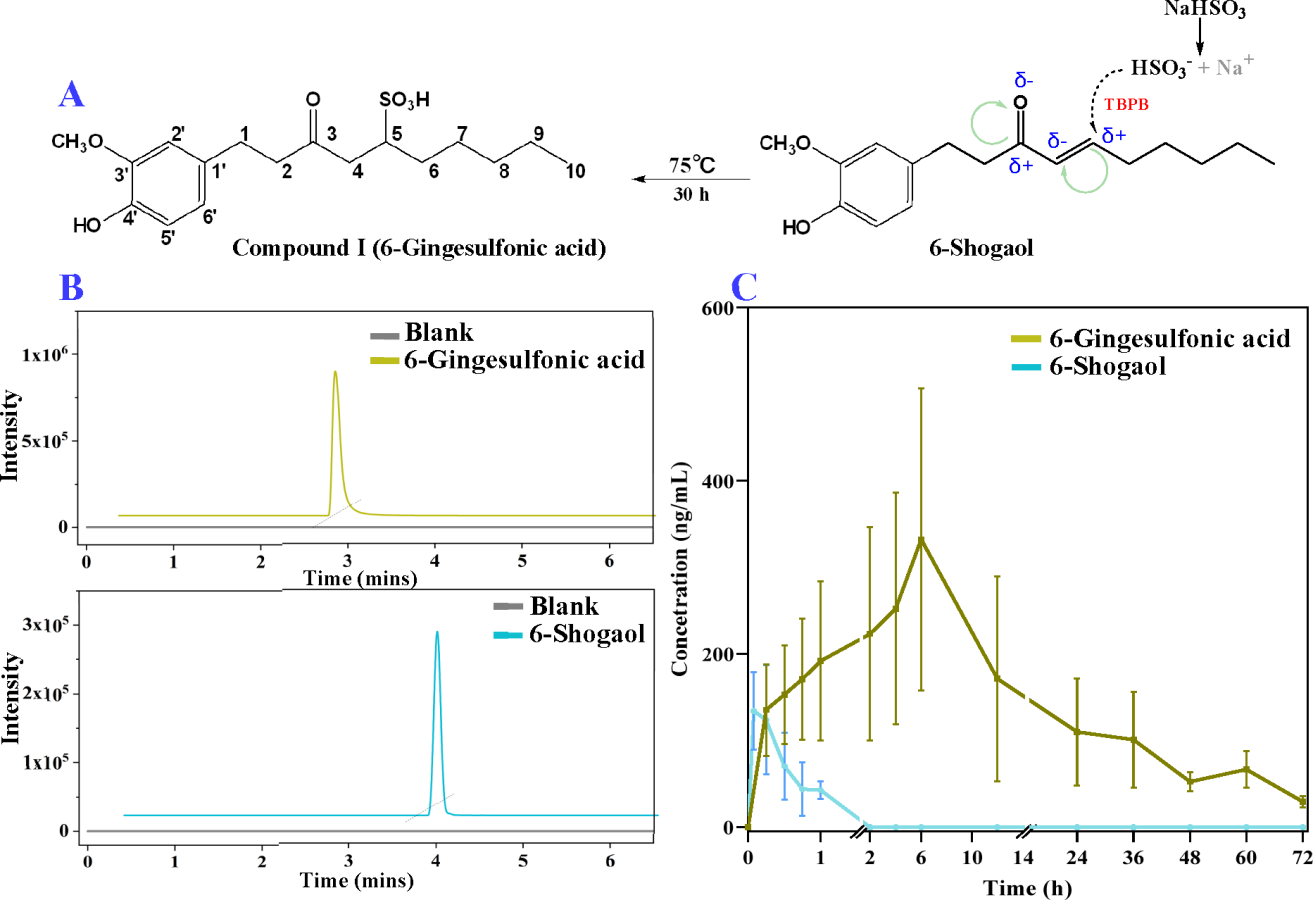
Structures and synthesis of compound I (6-gingesulfonic acid) (A), representative MRM chromatograms of 6-gingesulfonic acid and 6-shogaol in plasma (B), and the concentration–time profile in rat plasma after single oral administration (10 mg/kg) 6-gingesulfonic acid and 6-shogaol (*n* = 6) (C). (Blank: samples collected before the intragastric administration; 6-gingesulfonic acid: samples collected after single oral administration 10 mg/kg 6-gingesulfonic acid; 6-shogaol: sample collected after single oral administration 10 mg/kg 6-shogaol).

Compound I (Figure 1A) was obtained as a white amorphous powder. The molecular formula of C_17_H_26_O_6_S was established by high-resolution electrospray ionization mass spectrometry data (*m*/*z*: 357.1360 [M – H]^−^, calcd, 358.1450), indicative of five degrees of unsaturation. The ^1^H NMR spectrum (Table 1 and Figure S1) of I exhibited one methyl group (δ_H_ 0.89, t, *J* = 7.1 Hz), one methoxy group (δ_H_ 3.83, s), one thiomethine proton (δ_H_ 3.31, m), and three aromatic protons (δ_H_ 6.78, d, *J* = 1.9 Hz; 6.67, d, *J* = 8.0 Hz; 6.63, dd, *J* = 8.0, 1.9 Hz). Analysis of the ^13^C NMR and HSQC spectra of compound I (Table 1 and Figure S1) revealed 17 carbon signals, including one methyl, one methoxy (δ_C_ 56.36), six sp^3^ methylenes, one thiomethine (δ_C_ 57.09), one carbonyl carbon (δ_C_ 210.72) and a trisubstituted aromatic ring (δ_C_ 148.87, 145.68, 134.08, 121.73, 116.11, 113.12). One aromatic ring and one carbonyl group occupied five degrees of unsaturation, which required compound I to be monocyclic. Further analysis of ^1^H–^1^H COSY and TOCSY data (Figure S1) allowed the establishment of three spin systems: (a) H-1/H-2, (b) H-4/H-5/H-6/H-7/H-8/H-9/H-10, (c) H-5′/H-6′. The key HMBC (Figure S1) correlations from H-6′ to C-1/C-1′ and from H-2′ to C-1/C-1′ revealed the linkage of fragments (a) and (c) via C-1′, while those from H-2 to C-3/C-4, and from H-4 to C-2/C-3 fulfilled the linkage of fragments (a) and (b) through C-3, accomplishing the full assignment of the planar skeleton for compound I. Compared with the NMR data of 6-shogaol (Table 1),^13^ at the C4–C5 position, the NMR spectra of compound I showed signals for a saturated single bond (δ_H_ 2.52, 3.02, and 3.31; δ_C_ 44.80, 57.09) instead of the unsaturated keto-adjacent double bond (δ_H_ 6.12 and 6.90; δ_C_ 130.0, 148.7) of 6-shogaol. Compared with C5 (δ_C_ 148.7) in 6-shogaol, the observation of a higher chemical shift of C5 (δ_C_ 57.09)—a shift to a lower field—in compound I further elucidated that the sulfonyl group was attached to C-5. Based on these data, compound I was finally identified as 6-gingesulfonic acid by comparison with published literature NMR data (Table 1),^14^ which confirmed the speculation in our previous study.^9^

**Table 1:** NMR Data of Compound I, 6-Gingesulfonic Acid and 6-Shogaol (*J* in Hz)

	compound I (*m*/*z* 357.13) (CD_3_OD)		6-gingesulfonic acid^14^ (CD_3_OD)		6-shogaol^13^ (CD_3_OD)	
position	δ_C_, (101 MHz)	δ_H_, (400 MHz)	δ_C_, (125 MHz)	δ_H_, (500 MHz)	δ_C_, (150 MHz)	δ_H_, (600 MHz)
1	30.47	2.80, m	30.4	2.80, m	31.3	2.88
2	46.02	2.80, m	46.0	2.80, m	41.3	2.66
3	210.72		210.7		201.5	
4	44.80	2.52, dd (17.4, 6.5), 3.02, dd (17.5, 6.5)	44.8	2.50, dd (17.4, 6.4), 3.03, dd (17.4, 6.4)	130.0	6.12
5	57.09	3.31, m	57.1	3.30, m	148.7	6.90
6	32.02	1.43, 1.89, m	32.0	1.42, 1.90, m	32.1	2.22
7	27.94	1.31–1.36, m	27.9	1.30–1.35, m	27.6	1.48
8	33.00	1.31–1.36, m	33.0	1.30–1.35, m	29.8	1.34
9	23.54	1.31–1.36, m	23.5	1.30–1.35, m	22.1	1.34
10	14.42	0.89, t (7.1)	14.4	0.88, t (7.3)	13.0	0.93
1′	134.08		134.1		132.6	
2′	113.12	6.78, d (1.9)	113.1	6.77, d	111.8	
3′	148.87		148.9		147.6	
4′	145.68		145.7		144.5	
5′	116.11	6.67, d (8.0)	116.1	6.67, d	114.7	
6′	121.73	6.63, dd (8.0, 1.9)	121.7	6.60, dd	120.4	
O–CH_3_	56.36	3.83, s	56.4	3.82, s		3.84

### 3.2 Synthesis of 6-Gingesulfonic Acid

As aforementioned, we speculated that 6-gingesulfonic acid is transformed from 6-shogaol via electrophilic addition in the presence of sulfonic acid during sulfur fumigation.^9^ In order to further confirm the transformation mechanism, a method of chemical synthesis was developed based on a previous report with modifications.^15^ Allowing 6-shogaol to react with NaHSO_3_ in the prescence of TBPB (Figure 1A), then purifying the resulting residue by prep-TLC and prep-HPLC, produced the desired compound (14.2 mg, yield 54.8%). The chemical structure of the compound was verified as 6-gingesulfonic acid by mass spectra and NMR, and the purity of >98% was determined by peak area normalization. The successful synthesis of 6-gingesulfonic acid from 6-shogaol supports the speculation that 6-gingesulfonic acid is a sulfur fumigation-induced derivative of 6-shogaol. Moreover, the synthesis was much more efficient than the herbal extraction for preparing 6-gingesulfonic acid and therefore facilitated the following animal experiment by providing enough 6-gingesulfonic acid.

### 3.3 Quantitative Method Validations

The linear range of concentrations for 6-gingesulfonic acid in rat plasma was 0.320–500 ng/mL (*y* = 56.2282*x* – 30.0238) and the correlation coefficient(*r*^2^) was 0.9992. The linear range for 6-shogaol in rat plasma was 0.970–500 ng/mL (*y* = 6.3580*x* + 12.7310) with *r*^2^ = 0.9991 (*y* denotes the analytical response, and *x* denotes the concentrations). The LODs for 6-gingesulfonic acid and 6-shogaol were 0.0568 and 0.1465 ng/mL, and the LLOQs were 0.3086 and 0.4884 ng/mL, respectively. Accuracy and precision in analyses were all within ±15% (Table S1). The mean matrix effects for 6-gingesulfonic acid and 6-shogaol in plasma were 109.55 ± 11.85 and 98.92 ± 8.50% and the extraction recoveries were 90.45 ± 7.96 and 102.57 ± 6.61%, respectively (Table S2). The relative standard deviations of stability tests were all within ±15% (Table S3). The results indicated that the established UPLC–QQQ–MS/MS method was reliable and robust for the quantitative determination of 6-gingesulfonic acid and 6-shogaol in the pharmacokinetic analysis. The representative peak chromatograms (MRM) of plasma samples are shown in Figure 1B.

### 3.4 Comparison of the Pharmacokinetics of 6-Gingesulfonic Acid and 6-Shogaol

The mean plasma concentration versus time profiles for 6-gingesulfonic acid and 6-shogaol are presented graphically in Figure 1C, and the pharmacokinetic parameters are summarized in Table 2. As shown in Figure 1C and Table 2, after oral administration, 6-shogaol was rapidly absorbed into circulation with mean *C*_max_ at 154.60 ± 39.02 ng mL^–1^ and *T*_max_ at 0.14 ± 0.09 h; the AUC_0–∞_ was 120.61 ± 36.34 h ng mL^–1^. The Vz/F_obs, *T*_1/2_, MRT, and Cl/F_obs of 6-shogaol were 71.92 ± 33.03 μL kg^–1^, 0.64 ± 0.53 h, 1.02 ± 0.82 h, and 88.47 ± 22.71 h μL kg^–1^, respectively. The pharmacokinetic behavior of 6-shogaol was consistent with that in previous reports.^16^

**Table 2:** Pharmacokinetic Parameters of 6-Gingesulfonic Acid and 6-Shogaol (10 mg/kg) in Rats after Oral Administration^a^

parameters	6-gingesulfonic acid	6-shogaol	*p* value
*C*_max_ (ng mL^–1^)	519.75 ± 137.82	154.60 ± 39.02	0.000096
*T*_max_ (h)	5.33 ± 1.63	0.14 ± 0.09	0.000015
AUC_0–*t*_ (h ng mL^–1^)	8707.48 ± 1933.83	76.84 ± 26.89	<0.000001
AUC_0–∞_ (h ng mL^–1^)	9583.92 ± 2067.18	120.61 ± 36.34	<0.000001
Vz/F_obs (μL kg^–1^)	32.40 ± 9.94	71.92 ± 33.03	0.018571
*T*_1/2_ (h)	20.81 ± 5.23	0.64 ± 0.53	0.000003
MRT (h)	30.44 ± 1.83	1.02 ± 0.82	<0.000001
Cl/F_obs (h μL kg^–1^)	1.08 ± 0.19	88.47 ± 22.71	0.000003

a Data are presented as the mean ± SD (*n* = 6).

Compared to 6-shogaol, 6-gingesulfonic acid showed a longer *T*_max_ at 5.33 ± 1.63 h, a higher *C*_max_ at 519.75 ± 137.82 ng mL^–1^, and a larger AUC_0–∞_ at 9583.92 ± 2067.18 h ng mL^–1^ at significant levels, which demonstrated a much slower absorption rate but more of the prototype present in the blood. The lower Vz/F_obs of 6-gingesulfonic acid (32.40 ± 9.94 μL kg^–1^) indicated that it might be less widely distributed and/or tend to bind less often with biopolymers in vivo than 6-shogaol. In addition, 6-gingesulfonic acid appeared to be eliminated more slowly than 6-shogaol with a significantly longer *T*_1/2_ (20.81 ± 5.23 h) and MRT (30.44 ± 1.83 h), and a lower value of Cl/F_obs (1.08 ± 0.19 h μL kg^–1^).

The differences in pharmacokinetic behavior between 6-gingesulfonic acid and 6-shogaol should correlate strongly with their structural properties. With a sulfonic acid group, 6-gingesulfonic acid has a lower lipid–water partition coefficient and thus poorer liposolubility than 6-shogaol. This suggests more difficult transintestinal transportation and consequently a slower absorption rate than 6-shogaol. According to previous reports, orally administrated 6-shogaol was quickly absorbed in vivo to undergo the mercapturic acid pathway, and the conjugation reactions are favored to occur at the double bond beside the ketone group.^17,18^ However, since 6-gingesulfonic acid does not contain α, β-unsaturated ketone, conjugation reactions would be less likely to be involved in the in vivo biotransformation of 6-gingesulfonic acid. This prediction is further supported by the following metabolism investigation, in which metabolites of 6-gingesulfonic acid in the form of thiol-conjugated were relatively less detected than 6-shogaol, which may explain why more prototypes of 6-gingesulfonic acid were detected with a slower elimination rate than 6-shogaol.

### 3.5 Comparison of Metabolism of 6-Gingesulfonic Acid and 6-Shogaol

**Figure 2:**
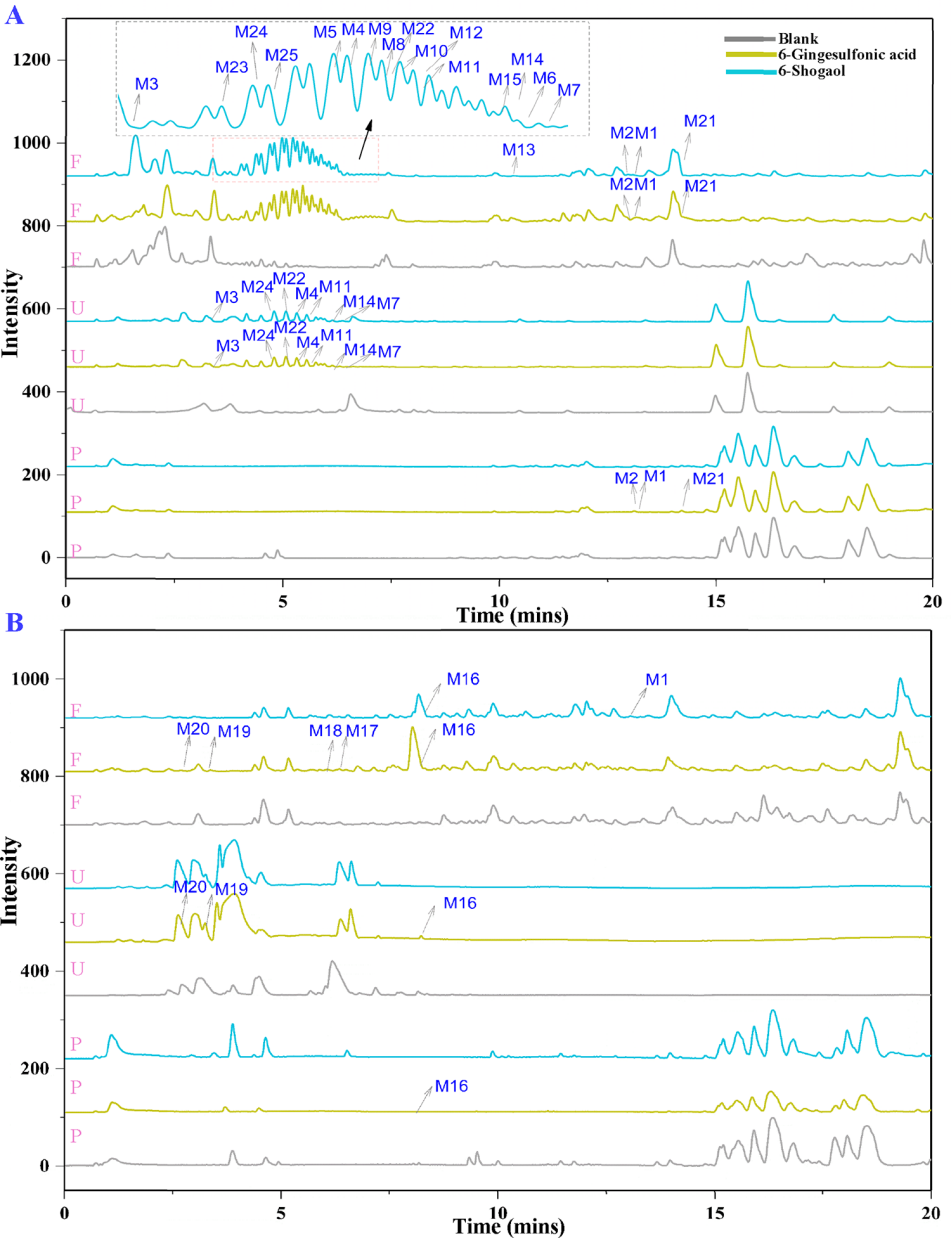
Representative BPCs of samples from control rats and rats treated with 10 mg/kg drugs at the time points of *T*_max_ in positive ion mode (A) and negative ion mode (B). (Blank: samples collected before the intragastric administration; 6-gingesulfonic acid: samples collected after single oral administration 10 mg/kg 6-gingesulfonic acid; 6-shogaol: sample collected after single oral administration 10 mg/kg 6-shogaol; F, U, and P represent rat feces, urine, and plasma samples, respectively).

The major metabolites of 6-gingesulfonic acid and 6-shogaol in rats were profiled by UPLC–QTOF–MS/MS, using both positive and negative ion modes. Representative base peak chromatograms (BPCs) for urine, feces, and plasma samples are shown in Figure 2. All metabolites from different biospecimens were qualitatively identified by comparing the elemental composition data determined from accurate molecular weight measurements and fragment ions with data of published known chemicals in our self-built library. Details of all metabolites identified are summarized in Table S4.

#### 3.5.1 Characterization of Metabolites of 6-Shogaol

**M1** showed 2 Da more and **M2** showed 12 Da less than 6-shogaol, they were tentatively identified as the demethylation metabolite 1-(4′-hydroxy-3′-methoxyphenyl)-4-decen-3-ol and hydrogenation metabolite 4-(3-hydroxydecyl)-1,2-benzenediol, respectively. **M3** was common and abundant in plasma, urine, and feces samples with the [M + H]^+^ ion at *m*/*z* 453.2129, which was 176 mass units higher than that of 6-shogaol, indicating **M3** was a glucuronidated 6-shogaol; it was specifically identified as *s*-6-shogaol-4′-O-β-glucuronide. This is consistent with a previous report that 6-shogaol was easily detected in the plasma in the form of glucuronide conjugates.^19^ The mass spectrum of metabolite **M4** exhibited the [M + H] ^+^ ion at *m*/*z* 584.2642, which was 307 Da higher than that of 6-shogaol, suggesting a glutathione (GSH) conjugate. Therefore, **M4** was identified as 5-glutathionyl-6-shogaol.^13^**M5** had the [M + H] ^+^ ion at *m*/*z* 586.2716, 2 Da higher than that of **M4**. It was identified as an isomer of reduced products on the carbonyl of **M4**, 5-glutathionyl-1-(4′-hydroxy-3′-methoxyphenyl)-4-decen-3-ol. **M6**, **M7,** and **M8** were assigned as cysteine conjugate metabolites,^20^ and **M9–M15** were metabolites that were involved in the mercapturic acid pathway.^20−22^ Furthermore, several isomers harboring a sulfonyl hydroxide were detected and were deduced to be the metabolites created by sulfated conjugation or sulfoconjugation. One of these (**M16**) was identified as 6-gingesulfonic acid confirmed by comparison with the standard.

#### 3.5.2 Characterization of Metabolites of 6-Gingesulfonic Acid

**Figure 3:**
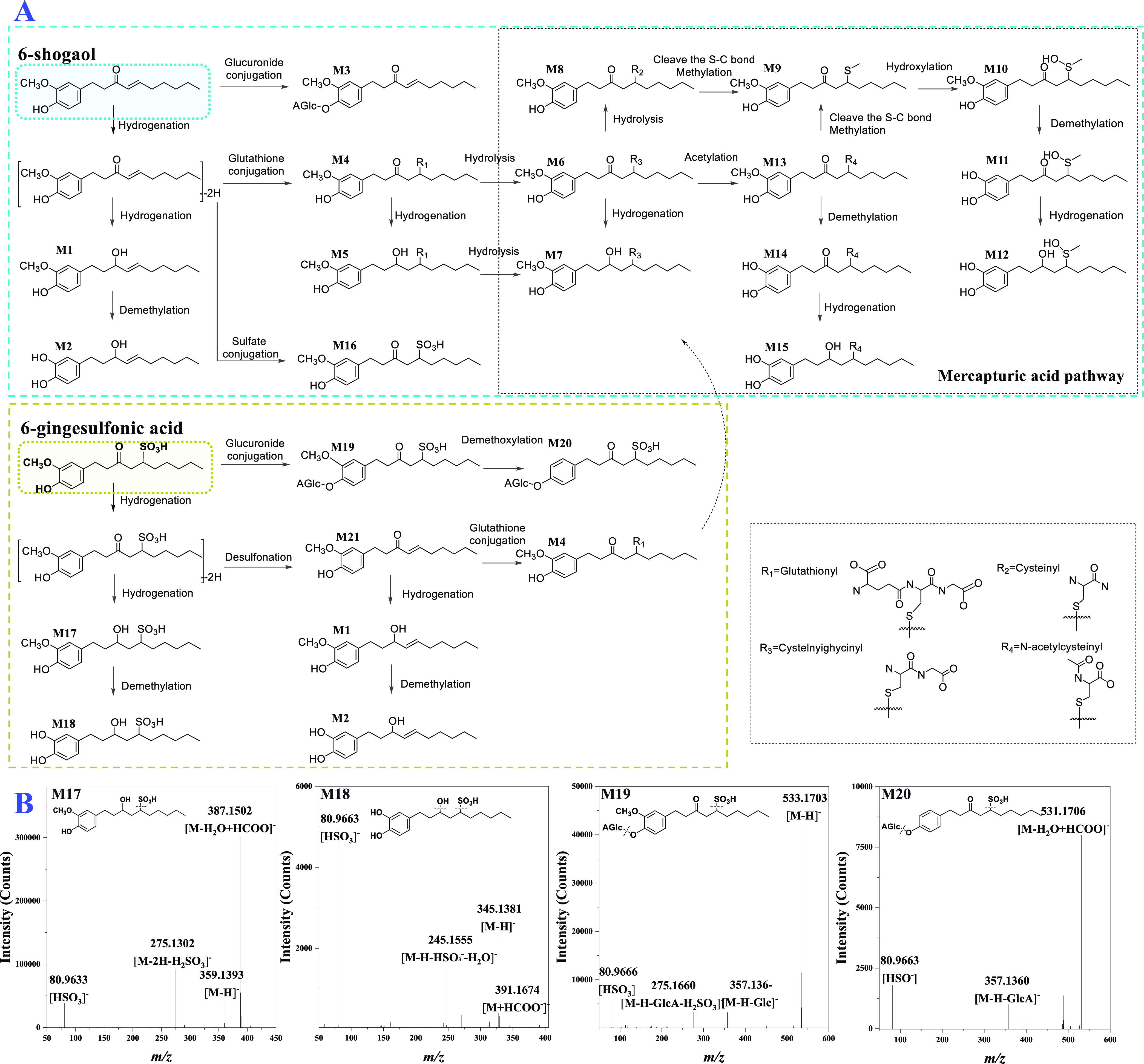
Proposed mechanisms for the metabolism of 6-gingesulfonic acid and 6-shogaol in rats (A) and MS/MS spectra of 4 new metabolites of 6-gingesulfonic acid (B).

**M17** had a molecular weight of 360.1606 as determined by its mass ions at *m*/*z* 359.1393 [M – H]^−^ and *m*/*z* 387.1502 [M + HCOO]^−^. This was 2 mass units higher than that of 6-gingesulfonic acid, demonstrating that **M17** was a hydrogenated metabolite of 6-gingesulfonic acid. The major fragment ions of **M17** were found at *m*/*z* 275.1302, 80.9633. This corresponds with the predicted molecular weight of 5-sulfonyl hydroxid-1-(4′-hydroxy-3′-methoxyphenyl)-4-decen-3-ol. The negative ion ESI–MS of **M18** displayed a molecular weight of 346.1461 as determined from *m*/*z* 345.1381 [M – H]^−^ and *m*/*z* 345.1381 [M – H – H_2_O]^−^ ion peak. This weight was 14 mass units lower than that of **M17** and it had abundant product ions at *m*/*z* 245.1555, 80.9663. The spectral features enabled us to tentatively identify **M18** as demethyl-5-sulfonyl hydroxid-1-(4′-hydroxy-3′-methoxyphenyl)-4-decen-3-ol. **M19** was 176 mass units higher than that of 6-gingesulfonic acid and had a molecular weight of 534.1775 as determined by the mass peak at *m*/*z* 533.1703 [M – H]^−^. This showed that **M19** was a glucuronide-conjugated metabolite of 6-gingesulfonic acid, a conclusion further supported by the fragment ions at *m*/*z* 357.1360, 275.1660, and 80.9666. Therefore, **M19** was tentatively identified as 6-gingesulfonic acid-4′-O-β-glucuronide. **M20** had a mass ion at *m*/*z* 531.1706 [M + HCOO^–^ – H_2_O]^−^ and a molecular weight of 504.1667, which was 147 mass units higher than that of 6-gingesulfonic acid and 30 mass units lower than that of **M19**. **M20** showed fragment ions at *m*/*z* 357.1360, 80.9663, suggesting that **M20** was a product of glucuronide-conjugated metabolite of 6-gingesulfonic acid by losing the methoxy group. **M20** was qualitatively identified as demethoxy-6-gingesulfonic acid-4′-O-β-glucuronide. In addition, it appears that 6-gingesulfonic acid could also undergo a desulfonation process and turn into **M21**, which was identified as 6-shogaol based on its fragment information and comparison with the standard. Its hydrogenation and demethylation products (**M1** and **M2**), glucuronidated product (**M3**), cysteine conjugate metabolites (**M6–M8**), and metabolites of the mercapturic acid pathway (**M9–M15**) were also found among the metabolites of 6-gingesulfonic acid. In addition, **M22–M25** was detected in both groups (Figure 3A and Table S4), also suggesting similar metabolic pathways of the two compounds.

The possible metabolic pathways for the main metabolites of 6-shogaol and 6-gingesulfonic acid are illustrated in Figure 3. In general, after oral administration, the two compounds went through a similar metabolic process including hydrogenation, demethylation, glucuronidation, and cysteine conjugation. More interestingly, they were transformed into each other in metabolism. Nevertheless, the metabolites in vivo of 6-shogaol and 6-gingesulfonic acid were qualitatively and quantitatively different, and such differences can be predicted to affect their bioactivities in vivo. For example, **M17–M20** were detected as the metabolites of 6-gingesulfonic acid but not found among the metabolites of 6-shogaol (Figure 3A,B). **M4** was a crucial precursor to mediate the mercapturic acid pathway.^23^**M4** was found in lower concentrations in urine and feces samples of 6-gingesulfonic acid than 6-shogaol. This may be at least partially because the unsaturated double bond in 6-shogaol facilitated its conjugation with glutathione to produce **M4**, whereas the sulfonic acid group in 6-gingesulfonic acid made it difficult for the reaction to proceed, which supports the pharmacokinetic results above. The cysteine-conjugated metabolite of 6-shogaol, **M8**, has been found to be bioactive in vivo and in vitro, presumably modifying multiple cysteine residues of Keap1 protein or inducing Nrf2 nuclear translocation.^24,25^ The ion intensity of **M8** was 2.90 × 10^4^ ± 293.5 in 6-shogaol but 9.26 × 10^3^ ± 619.4 in 6-gingesulfonic acid, with a statistical significance (with *p* < 0.05) in feces samples. This difference suggests that **M8** in the 6-gingesulfonic acid-treated group available for exerting the bioactivities could be significantly reduced.

### 3.6 Comparison of Antiproliferative Effects on Human Cancer Cells of 6-Gingesulfonic Acid and 6-Shogaol

**Figure 4:**
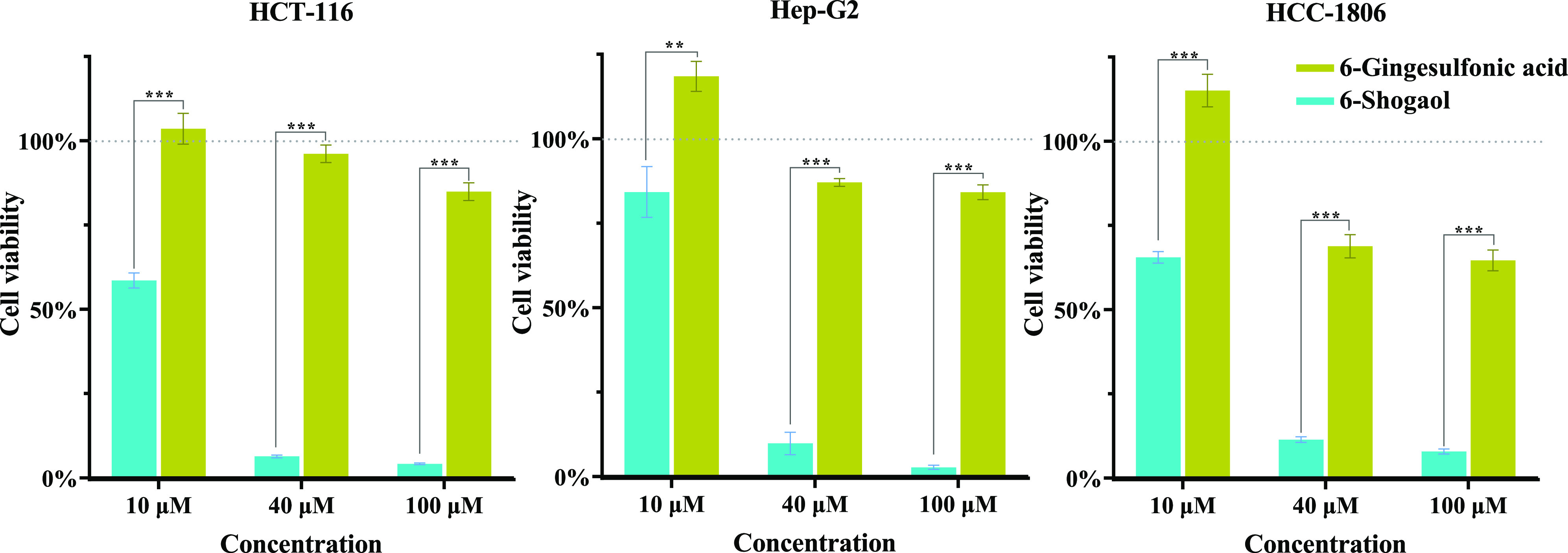
Viability of cells treated with 6-gingesulfonic acid and 6-shogaol (*n* = 6) (**, *p* < 0.01; ***, *p* < 0.001).

MTT assay was used to compare the bioactivity of 6-gingesulfonic acid and 6-shogaol in HCT-116, Hep-G2, and HCC-1806 cancer cells. The results are summarized in Figure 4. When treated with an increased concentration of 6-gingesulfonic acid or 6-shogaol, the cell viability decreased in a dose-dependent manner in all three cancer cell lines. At 10 μM dosing concentration, the viability of HCT-116 cells treated with 6-shogaol decreased to 58.57%, whereas those treated with 6-gingesulfonic acid showed no significant change compared with the control group. As the dosing concentration increased to 100 μM, the cell viability of HCT-116 cells decreased to 4.17% in the 6-shogaol treated group, compared with 84.89% living cells in the 6-gingesulfonic acid-treated group. Similar results appeared for the Hep-G2 and HCC-1806 cell lines: their cell viabilities were reduced drastically to 2.73 and 7.84%, respectively, by 100 μM 6-shogaol, however, the viability of cells treated with 6-gingesulfonic acid was 84.17 and 64.63%, respectively. These results indicated that 6-gingesulfonic acid exerted significantly weaker antiproliferative activity on tumor cells than 6-shogaol. Accumulating evidence has demonstrated that the α, β-unsaturated ketone in 6-shogaol is a critical functional group in the antitumor activities observed for the following reasons: (i) α, β-unsaturated ketone shows a strong hydrophobicity, which facilitates the cellular membrane transport of 6-shogaol.^5^ Further, its high electrophilicity means that, once 6-shogaol enters the tumor cell, it is prone to bind with the anticancer-related target protein;^26,27^ (ii) 6-shogaol acts as a Michael reaction acceptor to deplete intracellular GSH levels and then generate oxidative stress. These further releases mitochondria-associated apoptotic molecules to induce the apoptosis of tumor cells via the *p*53 pathway;^25^ and (iii) it is able to bind active redox sensor cysteines on Keap1 to repress Nrf2, which further suppresses the active transcription of various cytoprotective genes in tumor cells.^28^ However, α, β-unsaturated ketone is not part of the structure of 6-gingesulfonic acid; instead, a sulfonyl hydroxide attaches to the β site. Such structural differences should result in a stronger chemical polarity and thereby give 6-gingesulfonic acid weaker hydrophobicity than 6-shogaol.^29^ Consequently, 6-gingesulfonic acid would be more difficult to enter tumor cells by membrane transport than 6-shogaol. Moreover, since α and β sites of the ketone in 6-gingesulfonic acid are saturated, its electrophilicity is possibly lower than 6-shogaol. Hence, even if it can successfully enter tumor cells, 6-gingesulfonic acid may be less able to interact with the intracellular targets. All of these speculations warrant further investigation.

In conclusion, first compound I, which is generated in ginger by sulfur fumigation, was structurally confirmed as 6-gingesulfonic acid. Pharmacokinetics test in vivo showed that 6-gingesulfonic acid was more slowly absorbed and eliminated, with more prototypes existing in the blood than 6-shogaol. Metabolism profiling indicated that the two compounds shared similar metabolic processes but produced qualitatively and quantitatively different metabolites. It was further found that 6-gingesulfonic acid exerted significantly weaker antiproliferative activity on tumor cells than 6-shogaol. All these data provide not only insights into the impacts of sulfur fumigation ginger, but also chemical and biological evidence that sulfur fumigation may impair the healthcare functions of ginger.

## Supporting Information Available

^1^H NMR, ^13^C NMR, COSY NMR, TOCSY NMR, HSQC NMR, and HMBC NMR of 6-gingesulfonic acid, inter-day and intra-day assay accuracy and precision values, stability evaluation data of samples in rat plasma, and UPLC-QTOF-MS/MS data of major metabolites (PDF)

## Abbreviations used

NMR: nuclear magnetic resonance
TMS: tetramethylsilane
UPLC–QTOF–MS/MS: ultra-performance liquid chromatography with quadrupole time-of-flight mass spectrometer
TBPB: *tert*-butyl peroxybenzoate
prep-TLC: preparative thin-layer chromatography
prep-HPLC: preparative high-performance liquid chromatography
EDTA: ethylenediaminetetraacetic acid
*T*_max_: time when plasmatic concentrations reached a maximum value
QQQ: triple quadrupole
ESI: electrospray ion
LOD: limit of detection
LLOQ: lower limit of quantification
*C*_max_: maximum value of plasma concentrations
AUC: area under the curve
Vz/F_obs: apparent volume of distribution
*T*_1/2_: terminal half-life
MRT: mean residence time
Cl/F_obs: apparent total body clearance
SD: standard deviation
MTT: 3-(4,5-dimethylthiazol-2-yl)-2,5-diphenyltetrazolium bromide
DMSO: dimethyl sulfoxide
HR–ESI–MS: high-resolution electrospray ionization mass spectrometry
BPC: base peak chromatograms
MRM: multiple reaction monitoring
GSH: glutathione

## References

[ref1] Kubra I. R.; Rao L. J. M. An impression on current developments in the technology, chemistry, and biological activities of ginger (*Zingiber officinale* Roscoe). Crit. Rev. Food Sci. Nutr. 2012, 52, 651–688. 10.1080/10408398.2010.505689. 22591340

[ref2] Kou X.; Wang X.; Ji R.; Liu L.; Qiao Y.; Lou Z.; Ma C.; Li S.; Wang H.; Ho C. T. Occurrence, biological activity and metabolism of 6-shogaol. Food Funct. 2018, 9, 1310–1327. 10.1039/c7fo01354j. 29417118

[ref3] Mao Q. Q.; Xu X. Y.; Cao S. Y.; Gan R. Y.; Corke H.; Beta T.; Li H. B. Bioactive compounds and bioactivities of ginger (*Zingiber officinale* Roscoe). Foods 2019, 8, 185 10.3390/foods8060185. PMC6616534 31151279

[ref4] Semwal R. B.; Semwal D. K.; Combrinck S.; Viljoen A. M. Gingerols and shogaols: Important nutraceutical principles from ginger. Phytochemistry 2015, 117, 554–568. 10.1016/j.phytochem.2015.07.012. 26228533

[ref5] Pan M. H.; Hsieh M. C.; Hsu P. C.; Ho S. Y.; Lai C. S.; Wu H.; Sang G. S.; Ho C. T. 6-Shogaol suppressed lipopolysaccharide-induced up-expression of iNOS and COX-2 in murine macrophages. Mol. Nutr. Food Res. 2008, 52, 1467–1477. 10.1002/mnfr.200700515. 18683823

[ref6] Wu H.; Hsieh M. C.; Lo C. Y.; Liu C. B.; Sang S.; Ho C. T.; Pan M. H. 6-Shogaol is more effective than 6-gingerol and curcumin in inhibiting 12-*O*-tetradecanoylphorbol 13-acetate-induced tumor promotion in mice. Mol. Nutr. Food Res. 2010, 54, 1296–1306. 10.1002/mnfr.200900409. 20336681

[ref7] Ling H.; Yang H.; Tan S. H.; Chui W. K.; Chew E. H. 6-Shogaol, an active constituent of ginger, inhibits breast cancer cell invasion by reducing matrix metalloproteinase-9 expression via blockade of nuclear factor-κB activation. Br. J. Pharmacol. 2010, 161, 1763–1777. 10.1111/j.1476-5381.2010.00991.x. 20718733 PMC3010581

[ref8] Yan H.; Li P. H.; Zhou G. S.; Wang Y. J.; Bao B. H.; Wu Q. N.; Huang S. L. Rapid and practical qualitative and quantitative evaluation of non-fumigated ginger and sulfur-fumigated ginger via Fourier-transform infrared spectroscopy and chemometric methods. Food Chem. 2021, 341, 128241 10.1016/j.foodchem.2020.128241. 33038774

[ref9] Wu C. Y.; Kong M.; Zhang W.; Long F.; Zhou J.; Zhou S. S.; Xu J. D.; Xu J.; Li S. L. Impact of sulphur fumigation on the chemistry of ginger. Food Chem. 2018, 239, 953–963. 10.1016/j.foodchem.2017.07.033. 28873658

[ref10] United States Food and Drug Administration. Guidance for Industry, Bioanalytical Method Validation; U.S. Department of Health and Human Services, Food and Drug Administration Center for Drug Evaluation and Research (CDER): Washington, DC, USA, 2001.

[ref11] Zhang L.; Lin D.; Sun X.; Curth U.; Drosten C.; Sauerhering L.; Becker S.; Rox K.; Hilgenfeld R. Crystal structure of SARS-CoV-2 main protease provides a basis for design of improved α-ketoamide inhibitors. Science 2020, 368, 409–412. 10.1126/science.abb3405. 32198291 PMC7164518

[ref12] Zhang Y.; Huo M.; Zhou J.; Xie S. PKSolver: An add-in program for pharmacokinetic and pharmacodynamic data analysis in Microsoft Excel. Comput. Methods Programs Biomed. 2010, 99, 306–314. 10.1016/j.cmpb.2010.01.007. 20176408

[ref13] Chen H.; Lv L.; Soroka D.; Warin R. F.; Parks T. A.; Hu Y.; Zhu Y.; Chen X.; Sang S. Metabolism of [6]-shogaol in mice and in cancer cells. Drug Metab. Dispos. 2012, 40, 742–753. 10.1124/dmd.111.043331. 22246389 PMC3310425

[ref14] Hori Y.; Miura T.; Hirai Y.; Fukumura M.; Nemoto Y.; Toriizuka K.; Ida Y. Pharmacognostic studies on ginger and related drugs-part 1: five sulfonated compounds from *Zingiberis rhizome* (Shokyo). Phytochemistry 2003, 62, 613–617. 10.1016/S0031-9422(02)00618-0. 12560035

[ref15] Yoshikawa M.; Yamaguchi S.; Kunimi K.; Matsuda H.; Okuno Y.; Yamahara J.; Murakami N. Stomachic principles in ginger. III. An anti-ulcer principle, 6-gingesulfonic acid, and three monoacyldigalactosylglycerols, gingerglycolipids A, B, and C, from *Zingiberis Rhizoma* originating in Taiwan. Chem. Pharm. Bull. 1994, 42, 1226–1230. 10.1248/cpb.42.1226. 8069973

[ref16] Asami A.; Shimada T.; Mizuhara Y.; Asano T.; Takeda S.; Aburada T.; Miyamoto K. .; Aburada M. Pharmacokinetics of [6]-shogaol, a pungent ingredient of *Zingiber officinale* Roscoe (Part I). J. Nat. Med. 2010, 64, 281–287. 10.1007/s11418-010-0404-y. 20238179

[ref17] Wang P.; Wang R.; Zhu Y.; Sang S. Interindividual variability in metabolism of [6]-shogaol by gut microbiota. J. Agric. Food Chem. 2017, 65, 9618–9625. 10.1021/acs.jafc.7b02850. 29019244

[ref18] Chen H.; Soroka D. N.; Hu Y.; Chen X.; Sang S. Characterization of thiol-conjugated metabolites of ginger components shogaols in mouse and human urine and modulation of the glutathione levels in cancer cells by [6]-shogaol. Mol. Nutr. Food Res. 2013, 57, 447–458. 10.1002/mnfr.201200679. 23322393 PMC3817846

[ref19] Zick S. M.; Djuric Z.; Ruffin M. T.; Litzinger A. J.; Normolle D. P.; Alrawi S.; Feng M. R.; Brenner D. E. Pharmacokinetics of 6-gingerol, 8-gingerol, 10-gingerol, and 6-shogaol and conjugate metabolites in healthy human subjects. Cancer Epidemiol., Biomarkers Prev. 2008, 17, 1930–1936. 10.1158/1055-9965.epi-07-2934. 18708382 PMC2676573

[ref20] Chen H.; Sang S. Identification of phase II metabolites of thiol-conjugated [6]-shogaol in mouse urine using high-performance liquid chromatography tandem mass spectrometry. J. Chromatogr. B 2012, 907, 126–139. 10.1016/j.jchromb.2012.09.020. PMC3480212 23031413

[ref21] He L.; Qin Z.; Li M.; Chen Z.; Zeng C.; Yao Z.; Yu Y.; Dai Y.; Yao X. Metabolic profiles of ginger, a functional food, and its representative pungent compounds in rats by ultraperformance liquid chromatography coupled with quadrupole time-of-flight tandem mass spectrometry. J. Agric. Food Chem. 2018, 66, 9010–9033. 10.1021/acs.jafc.8b03600. 30068078

[ref22] Tang W.; Abbott F. S. Characterization of thiol-conjugated metabolites of 2-propylpent-4-enoic acid (4-enevpa), a toxic metabolite of valproic acid, by electrospray tandem mass spectrometry. J. Mass Spectrom. 1996, 31, 926–936. 10.1002/(sici)1096-9888(199608)31:8<926::aid-jms383>3.0.co;2-p. 8799319

[ref23] Chen H.; Soroka D. N.; Zhu Y.; Hu Y.; Chen X.; Sang S. Cysteine-conjugated metabolite of ginger component [6]-shogaol serves as a carrier of [6]-shogaol in cancer cells and in mice. Chem. Res. Toxicol. 2013, 26, 976–985. 10.1021/tx4001286. 23638641 PMC3767927

[ref24] Chen H.; Fu J.; Chen H.; Hu Y.; Soroka D. N.; Prigge J. R.; Schmidt E. E.; Yan F.; Major M. B.; Chen X.; Sang S. Ginger compound [6]-shogaol and its cysteine-conjugated metabolite (M2) activate Nrf2 in colon epithelial cells in vitro and in vivo. Chem. Res. Toxicol. 2014, 27, 1575–1585. 10.1021/tx500211x. 25148906 PMC4176387

[ref25] Warin R. F.; Chen H.; Soroka D. N.; Zhu Y.; Sang S. Induction of lung cancer cell apoptosis through a *p*53 pathway by [6]-shogaol and its cysteine-conjugated metabolite M2. J. Agric. Food Chem. 2014, 62, 1352–1362. 10.1021/jf405573e. 24446736 PMC3983336

[ref26] Jacob J. N. Comparative studies in relation to the structure and biochemical properties of the active compounds in the volatile and nonvolatile fractions of turmeric (*C. longa*) and ginger (*Z. officinale*). Stud. Nat. Prod. Chem. 2016, 48, 101–135. 10.1016/B978-0-444-63602-7.00004-7.

[ref27] Prasad S.; Tyagi A. K. Ginger and its constituents: role in prevention and treatment of gastrointestinal cancer. Gastroenterol. Res. Pract. 2015, 2015, 142979 10.1155/2015/142979. 25838819 PMC4369959

[ref28] Liu Q.; Peng Y. B.; Zhou P.; Qi L. W.; Zhang M.; Gao N.; Liu E.; Li P. 6-Shogaol induces apoptosis in human leukemia cells through a process involving caspase-mediated cleavage of eIF2α. Mol. Cancer 2013, 12, 135 10.1186/1476-4598-12-135. 24215632 PMC4176122

[ref29] Saha A.; Blando J.; Silver E.; Beltran L.; Sessler J.; DiGiovanni J. 6-Shogaol from dried ginger inhibits growth of prostate cancer cells both in vitro and in vivo through inhibition of STAT3 and NF-κB signaling. Cancer Prev. Res. 2014, 7, 627–638. 10.1158/1940-6207.capr-13-0420. 24691500

