# Supplementary material for: Impact of Sulfur Fumigation on Ginger: Chemical and Biological Evidence
Source: J Agric Food Chem. 2022 Sep 21;70(39):12577–86. doi: 10.1021/acs.jafc.2c05710 (PMC9545147; doi:10.1021/acs.jafc.2c05710)
Supplement: Supplementary file 1 — jf2c05710_si_001.pdf [file jf2c05710_si_001.pdf]

Supplementary Material

Impact of sulfur fumigation on ginger: Chemical and biological evidence

Wei-Hao Zhang [a, #], Han-Yan Luo [a, #], Jing Fang [a], Chen-Liang Zhao [b], Kam-Chun Chan [a], Yui-Man Chan [a], Cai-Xia Dong [c], Hu-Biao Chen [a], Zhong-Zhen Zhao [a], Song-Lin Li [d, *], Jun Xu [a, d, *]

[a] *School of Chinese Medicine, Hong Kong Baptist University, Hong Kong*

[b] *College of Pharmacy, Guizhou University of Traditional Chinese Medicine, Guizhou 550002, China*

[c] *Tianjin Key Laboratory on Technologies Enabling Development of Clinical Therapeutics and Diagnosis, School of Pharmacy, Tianjin Medical University, Tianjin 300070, China*

[d] *Department of Pharmaceutical Analysis, Affiliated Hospital of Integrated Traditional Chinese and Western Medicine, Nanjing University of Chinese Medicine, Nanjing 210028, China*

*Corresponding author:

Song-Lin Li, Ph D

Department of Pharmaceutical Analysis, Hospital of Integrated Traditional Chinese and Western Medicine

Affiliated to Nanjing University of Chinese Medicine, Nanjing 210028, PR China

Tel: 86-25-85639640, E-mail: songlinli64@126.com (S.-L. Li)

Jun Xu, Ph D

School of Chinese Medicine, Hong Kong Baptist University, Hong Kong

Tel: 852-34112423, E-mail: davidxujun@hkbu.edu.hk (J. Xu)

Figure S1. NMR spectra of 6-gingesulfonic acid

^{1}H NMR of 6-gingesulfonic acid

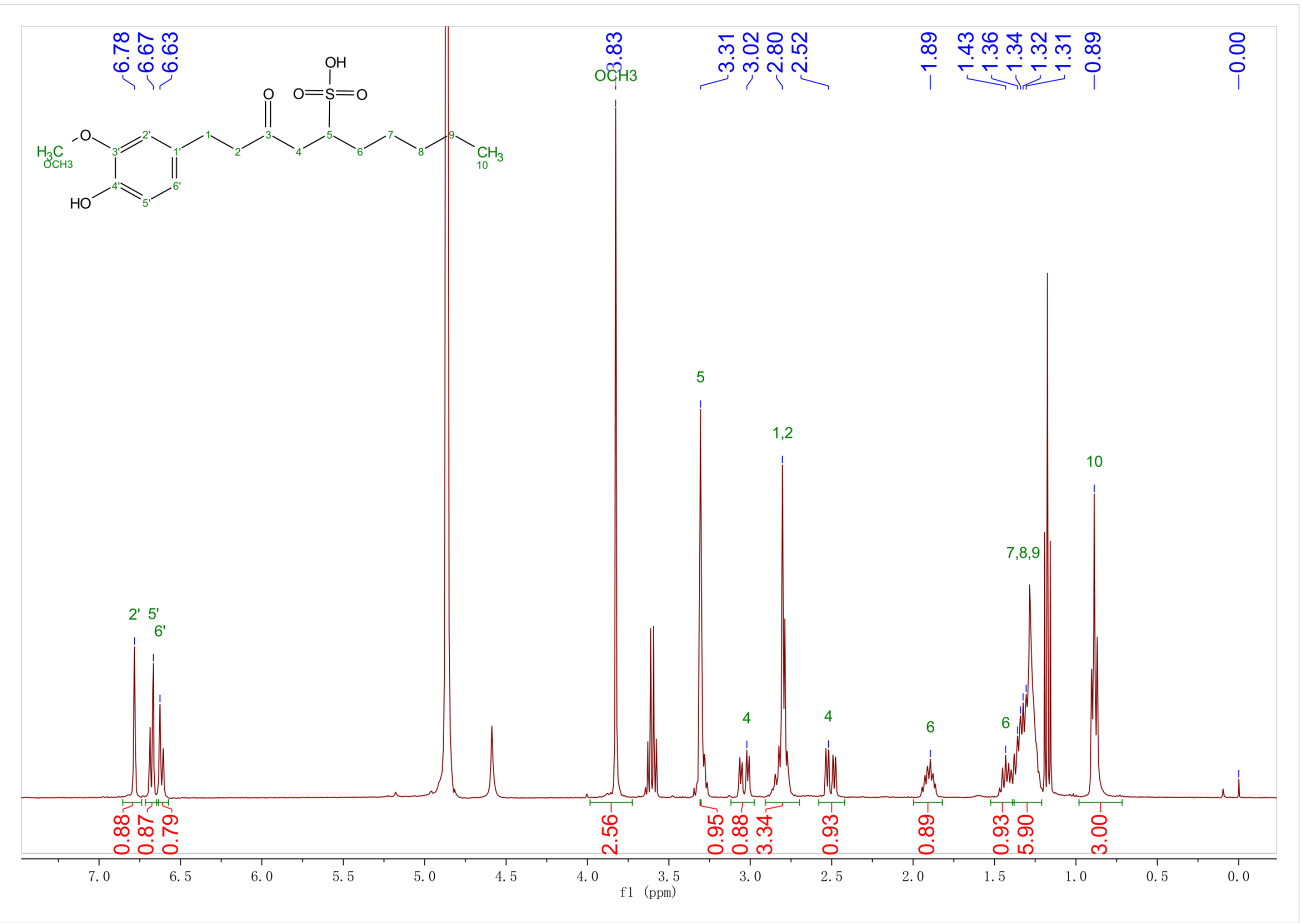

^{13}C NMR of 6-gingesulfonic acid

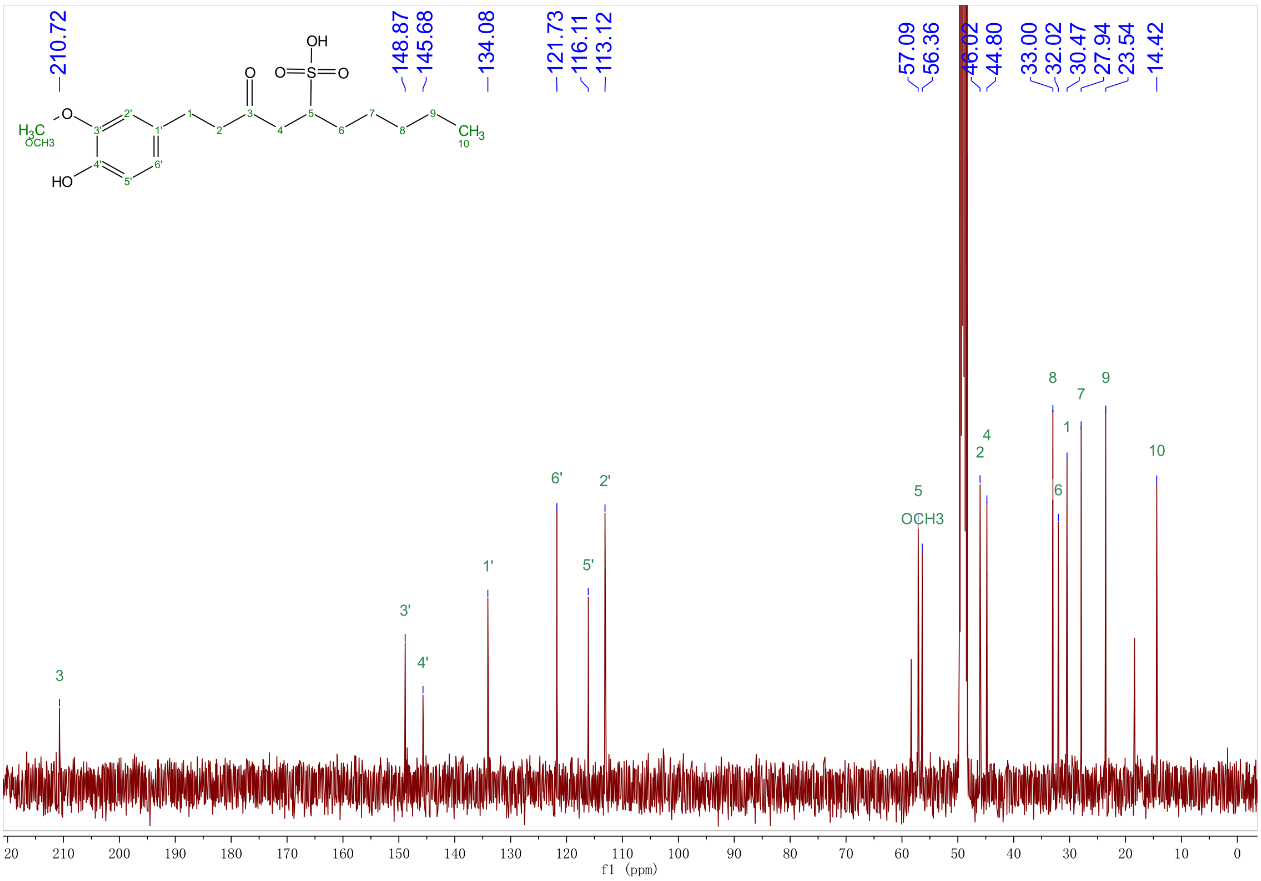

COSY NMR of 6-gingesulfonic acid

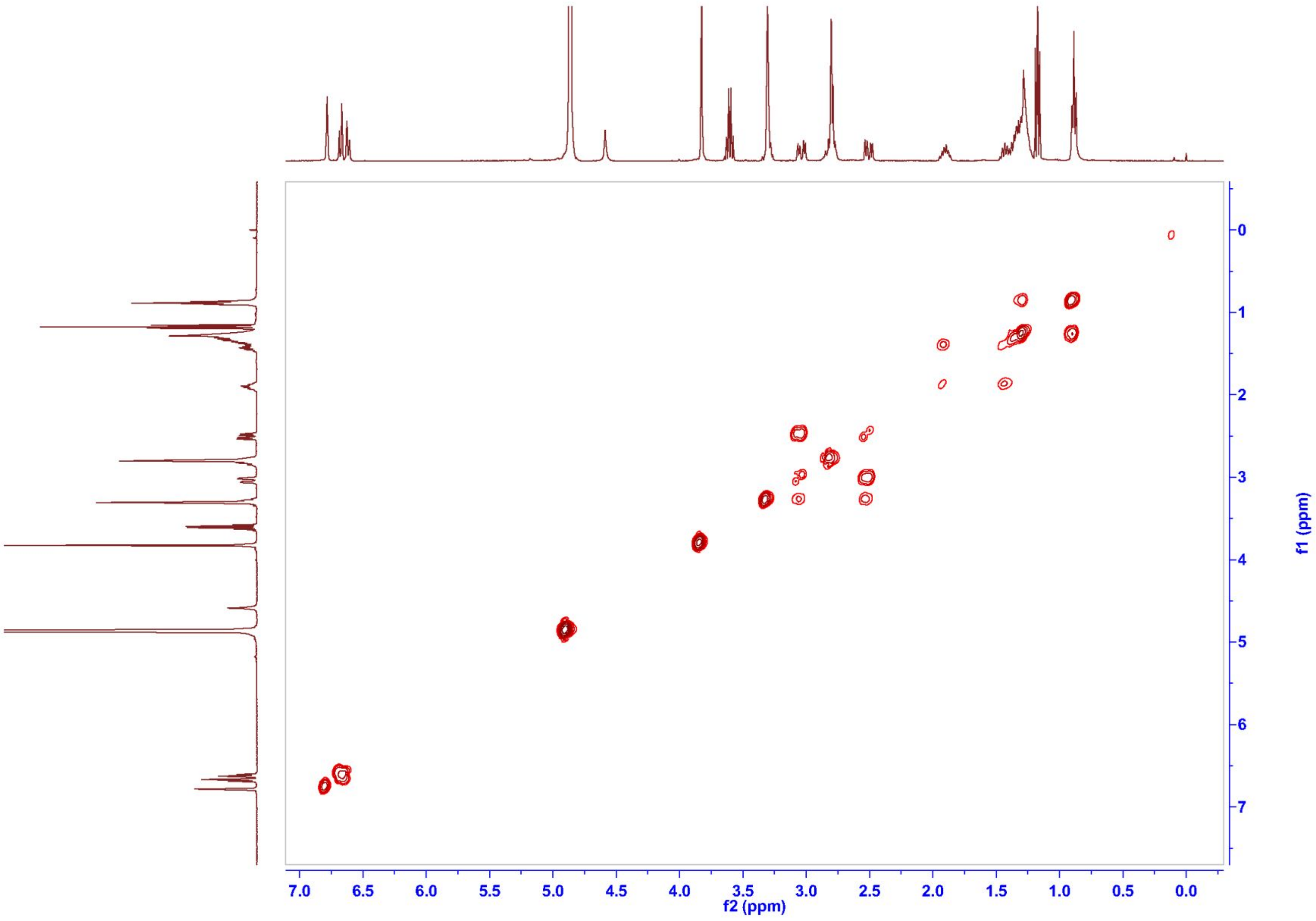

TOCSY NMR of 6-gingesulfonic acid

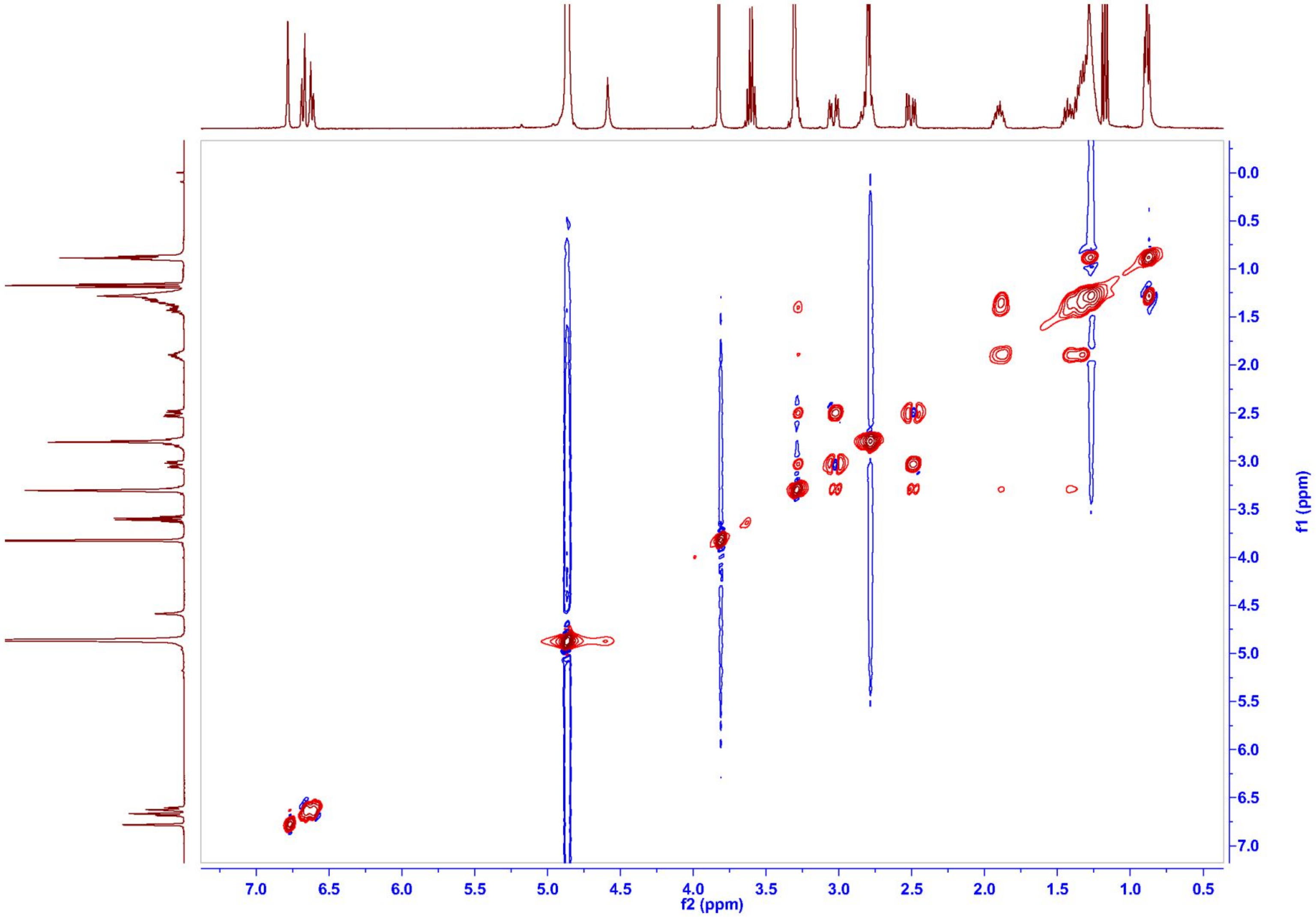

HSQC NMR of 6-gingesulfonic acid

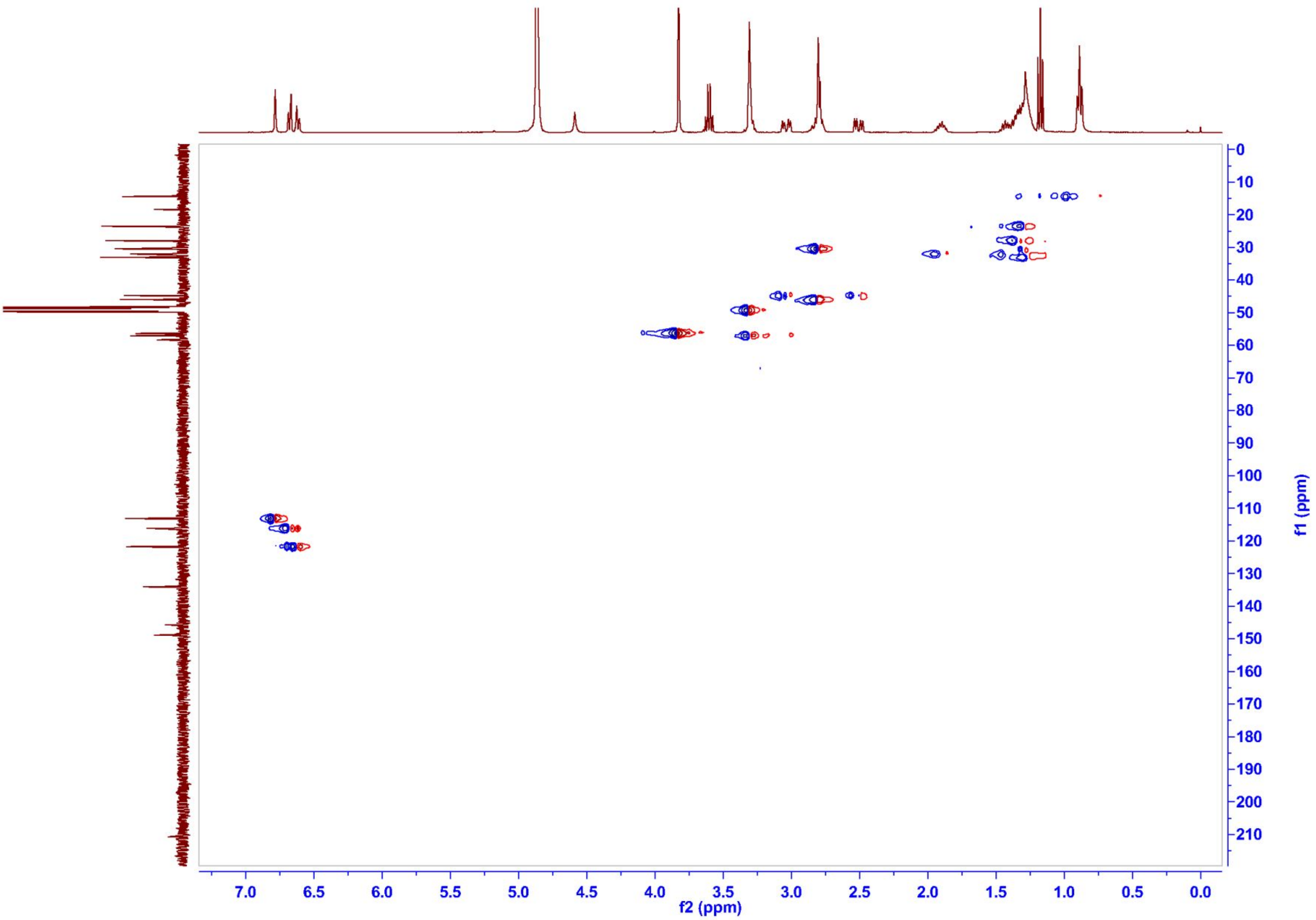

HMBC NMR of 6-gingesulfonic acid

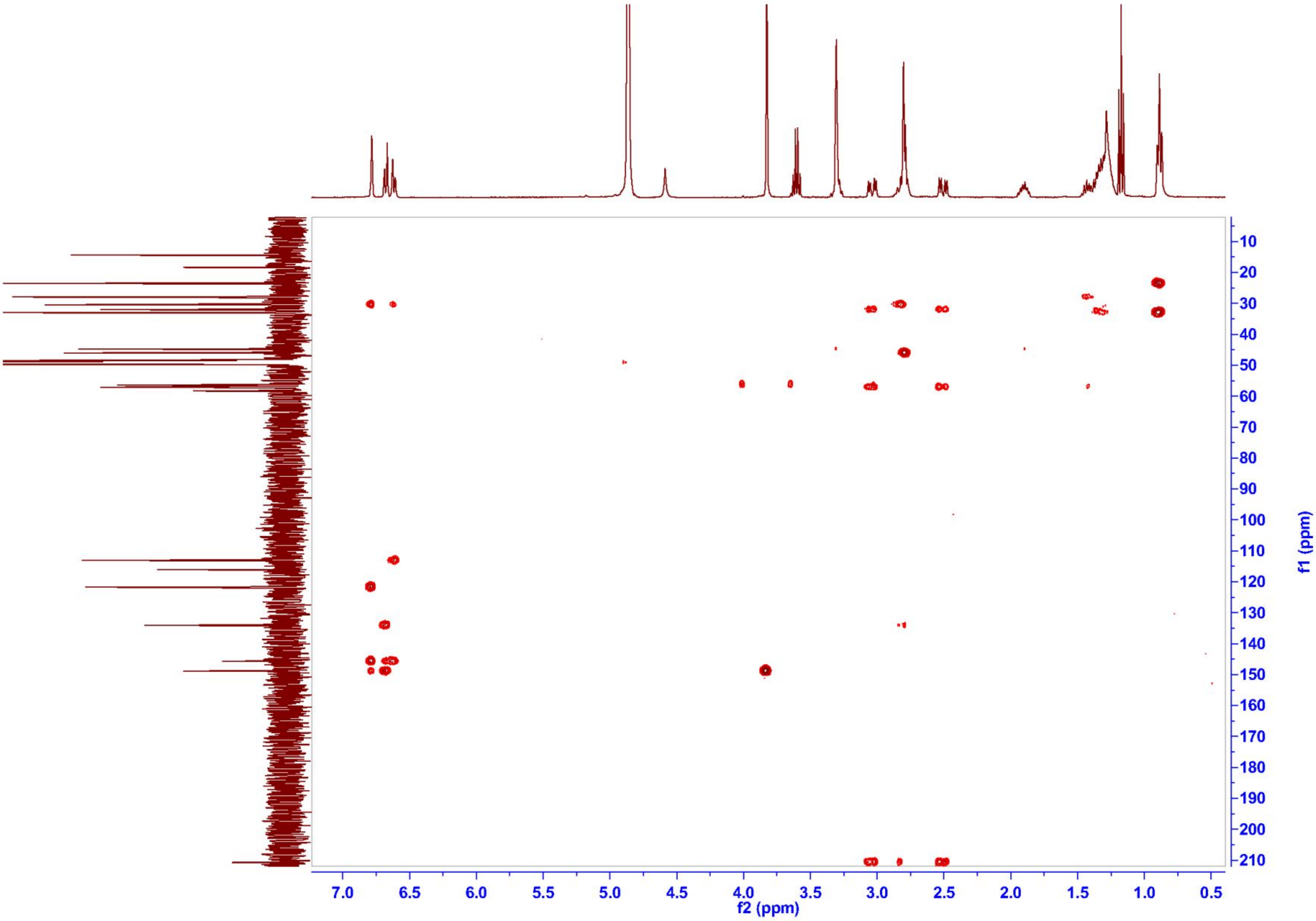

Table S1. Inter-day and intra-day assay accuracy (% Bias) and precision (% RSD) values of UPLC-QQQ-MS/MS method for the determination in rat plasma

	Intra-day				Inter-day		
	Nominal concentration (ng/mL)	Observed concentration (ng/mL)	Bias	RSD	Observed concentration (ng/mL)	Bias	RSD
**6-gingesulfonic acid**							
	2.00	1.82±0.08	-8.89%	4.24%	1.86±0.14	-6.68%	7.27%
	4.00	4.19±0.39	4.92%	9.26%	4.19±0.32	4.89%	7.70%
	20.00	18.41±1.13	-7.97%	6.16%	18.61±1.19	-6.95%	6.43%
	100.00	96.58±8.35	-3.42%	8.65%	106.10±5.61	6.10%	5.29%
	250.00	255.27±12.69	2.11%	4.97%	241.65±13.85	-3.34%	5.73%
	500.00	502.19±17.64	4.44%	3.53%	489.17±4.36	-2.16%	0.89%
**6-shogaol**							
	3.91	3.64±0.14	-6.90%	3.80%	3.63±0.27	-6.87%	7.52%
	7.81	8.14±0.23	4.16%	2.85%	8.28±0.70	6.10%	8.54%
	15.63	17.29±1.24	10.71%	7.14%	16.54±2.35	5.84%	14.19%
	31.25	32.82±3.59	5.03%	10.93%	27.18±0.76	-13.02%	2.80%
	62.50	64.98±5.11	3.98%	7.87%	64.11±1.16	2.57%	1.82%
	125.00	129.98±10.23	3.98%	7.87%	125.26±0.77	0.21%	0.62%

Data are presented as the mean ± SD (n=3)

Table S2. Matrix effect (ME) and recovery (RE) data in rat plasma

	Set1 (ng/mL)	Set2 (ng/mL)	Set3 (ng/mL)	ME	RE
**6-gingesulfonic acid**					
	0.55±0.14	0.57±0.32	0.49±0.24	103.77%	86.93%
	2.68±0.19	2.72±0.15	2.17±0.57	101.36%	79.81%
	5.62±0.51	5.58±0.52	4.95±0.65	99.37%	88.83%
	12.15±0.71	15.29±0.83	15.03±0.62	115.90%	98.34%
	35.32±0.69	44.99±1.55	44.25±0.60	127.37%	98.37%
			**Average**	109.55±11.85%	90.45±7.96%
**6-shogaol**					
	3.43±0.27	3.10±0.41	2.83±0.39	90.29%	91.20%
	12.62±0.37	13.05±0.67	13.83±0.46	103.44%	105.99%
	39.64±0.58	33.18±0.38	34.78±0.75	93.70%	104.83%
	143.06±2.03	159.44±1.18	164.08±4.58	111.45%	102.91%
	928.79±4.19	859.83±2.36	927.85±3.90	95.75%	107.91%
			**Average**	98.92±8.50%	102.57±6.61%

Data are presented as the mean ± SD (n=3)

Table S3. The stability evaluation data of samples in rat plasma

		Low (ng/mL)	Medium (ng/mL)	High (ng/mL)	Stability (Mean±SD)
**6-gingesulfonic acid**					
	Origin	82.74±0.13	342.03±0.31	711.73±1.54	
	Freeze and Thaw	72.63±0.69	328.68±1.02	668.42±0.58	92.6±4.31%
	Short Term	81.50±0.31	345.70±0.53	698.97±1.03	99.26±1.58%
	Long Term	75.78±0.29	302.18±0.78	689.15±1.76	92.26±4.28%
	Postpreparative	76.73±0.42	351.41±1.49	705.25±1.84	98.19±5.06%
**6-shogaol**					
	Origin	36.35±0.21	77.50±1.05	219.84±1.27	
	Freeze and Thaw	38.58±0.33	68.61±0.59	205.50±1.81	96.06±9.07%
	Short Term	33.29±0.37	81.61±0.86	199.44±1.27	95.88±8.18%
	Long Term	37.84±0.22	67.48±0.77	247.33±0.59	101.23±12.95%
	Postpreparative	37.08±39	66.59±0.52	219.13±0.92	95.87±8.69%

Data are presented as the mean ± SD (n=3)

Table S4. UPLC-QTOF-MS/MS Data of Major Metabolites of 6-gingesulfonic acid and 6-shogaol in Rat Biological Samples

Metabolism	Rt (min)	Elemental Composition	Compound name	Theoretical mass	Measured mass	ppm	Selected ion	MS² fragmentation	6-Gingesulfonic acid treated	6-Shogaol treated
M1[24]	13.329	$C_{17}H_{28}O_3$	1-(4′-hydroxy-3′-methoxyphenyl)-4-decen-3-ol	280.1906	325.2027	0.65	$[M+HCOO]^-$	262.1920, 137.0608	F	FUP
M2[24]	13.147	$C_{16}H_{26}O_3$	4-(3-Hydroxydecyl)-1,2-benzenediol	266.1887	267.1967	2.01	$[M+H]^+$	249.1855, 123.9344	F	FUP
M3[20, 22]	3.495	$C_{23}H_{32}O_9$	*s*- 6-shogaol-4′-O-*β*-glucuronide	452.2049	453.2129	-0.31	$[M+H]^+$	275.1660, 175.0236	FU	FUP
M4[24]	5.063	$C_{27}H_{41}N_3O_9S$	5-glutathionyl- 6-shogaol	583.2565	584.2642	0.32	$[M+H]^+$	308.1381, 162.0227	FU	FU
M5[21]	4.976	$C_{27}H_{43}N_3O_9S$	5-glutathionyl-1-(4′-hydroxy-3′-methoxyphenyl)-4-decen-3-ol	585.2798	586.2716	0.18	$[M+H]^+$	439.2263, 163.0753	F	F
M6[19, 22]	6.496	$C_{22}H_{36}N_2O_6S$	5-cysteinylglycinyl-6-shogaol	456.2296	437.2113	-1.01	$[M-H_2O+H]^+$	439.1809, 163.0817	F	F
M7[19, 22]	6.38	$C_{22}H_{38}N_2O_6S$	5-cysteinylglycinyl-1-(4′-hydroxy-3′-methoxyphenyl)-4-decen-3-ol	458.2607	459.2368	0.55	$[M+H]^+$	422.1323, 177.0751	FU	FU
M8[19, 22]	5.163	$C_{20}H_{31}NO_5S$	5-cysteinyl-6-shogaol	397.1954	397.1880	-0.17	M^+	277.9349, 137.1315	F	F
M9[19, 22]	5.021	$C_{20}H_{33}NO_5S$	5-cysteinyl-1-(4′-hydroxy-3′-methoxyphenyl)-4-decen-3-ol	399.2093	399.2157	3.4	M^+	263.1720, 137.1608	F	F
M10[19, 22]	5.327	$C_{18}H_{28}O_4S$	3'-Decanone, 1-(4-hydroxy-3-methoxyphenyl)-5-(methylthio)-sulfoxide	340.1712	341.1739	-1.19	$[M+H]^+$	276.1177, 135.0877	F	F
M11[19, 22]	5.561	$C_{17}H_{26}O_4S$	Monodemethylated-3-Decanone, 1-(4-hydroxy-3-methoxyphenyl)-5-(methylthio)-sulfoxide	326.1518	327.1604	0.84	$[M+H]^+$	280.2056, 100.9350	FU	FU
M12[19, 22]	5.474	$C_{17}H_{28}O_4S$	3-ketone-Monodemethylated-3-Decanone, 1-(4-hydroxy-3-methoxyphenyl)-5-(methylthio)-sulfoxide	328.1717	329.1639	2.73	$[M+H]^+$	311.1692, 247.6828	F	F
M13[19, 22]	11.207	$C_{22}H_{33}NO_6S$	5-*N*-acetylcysteinyl-6-shogaol	439.1948	440.2113	3.21	$[M+H]^+$	398.1998,	F	F

								363.1630		
M14[19, 22]	6.204	$C_{21}H_{31}NO_6S$	3′-demethylated-5-*N*-acetylcysteinyl-6-shogaol	425.1970	426.1955	-2.31	$[M+H]^+$	263,245,123	FU	FU
M15[19, 22]	6.147	$C_{21}H_{33}NO_6S$	3-ketone-3′-demethylated-5-*N*-acetylcysteinyl- 6-shogaol	427.2005	409.1898	-4.59	$[M-H_2O]^+$	263.1043, 149.0945	F	F
M16	8.043	$C_{17}H_{27}O_6S$	6-gingesulfonic acid	358.1450	357.1360	-0.35	$[M-H]^-$	257.1306 139.1138	FUP	FU
M17	8.250	$C_{17}H_{28}O_6S$	5-sulfonyl hydroxid-1-(4′-hydroxy-3′-methoxyphenyl)-4-decen-3-ol	360.1606	387.1502	-0.13	$[M-H_2O+HCOO]^-$	359.1393, 257.1302	F	--
M18	7.779	$C_{16}H_{26}O_6S$	Demethyl-5-sulfonyl hydroxid-1-(4′-hydroxy-3′-methoxyphenyl)-4-decen-3-ol	346.1461	345.1381	0.93	$[M-H]^-$	245.1555, 161.0279,	F	--
M19	2.674	$C_{23}H_{34}O_{12}S$	6-gingesulfonic acid-4′-O-*β*-glucuronide	534.1775	533.1703	0.84	$[M-H]^-$	357.1360, 80.9666	FU	--
M20	2.988	$C_{22}H_{32}O_{11}S$	Demethoxy-6-gingesulfonic acid-4′-O-*β*-glucuronide	504.1667	531.1706	0.34	$[M-H_2O+HCOO]^-$	257.1306, 80.9663	FU	--
M21	14.020	$C_{17}H_{24}O_3$	6-shogaol	276.1751	277.1665	0.36	$[M+H]^+$	259.1305, 137.5342	FU	FUP
M22[22]	5.239	$C_{18}H_{30}O_4S$	3-ketone-3-Decanone, 1-(4-hydroxy-3-methoxyphenyl)-5-(methylthio)-sulfoxide	342.1887	343.1751	0.42	$[M+H]^+$	325.1792, 177.1694	FU	FU
M23[22]	4.156	$C_{22}H_{35}NO_6S$	5-*N*-acetylcysteinyl-1-(4′-hydroxy-3′-methoxyphenyl)-4-decen-3-ol	441.2167	442.2259	-4.11	$[M+H]^+$	311.1692, 263.1660	F	F
M24[22]	4.507	$C_{24}H_{38}O_9S$	Methylthio glucuronide 5-glutathionyl-6-shogaol	502.2217	507.1862	-2.87	$[M-H_2O+Na]^+$	484.1108, 137.5542	FU	FU
M25[22]	4.39	$C_{24}H_{36}O_9S$	Methylthio glucuronide 5-glutathionyl-1-(4′-hydroxy-3′-methoxyphenyl)-4-decen-3-ol	500.2065	523.2408	1.31	$[M+Na]^+$	327.1994, 177.0914	F	F

F, U and P represent rat feces, urine and plasma samples, respectively.
